# Supplementary material for: A molecular atlas of innate immunity to adjuvanted and live attenuated vaccines, in mice
Source: Nat Commun. 2022 Jan 27;13:549. doi: 10.1038/s41467-022-28197-9 (PMC8795432; doi:10.1038/s41467-022-28197-9)
Supplement: Supplementary file 1 — Supplementary Information [file 41467_2022_28197_MOESM1_ESM.pdf]

A Molecular Atlas of Innate Immunity to Adjuvanted and Live Attenuated Vaccines, in Mice

Audrey Lee[1,*], Madeleine KD Scott[1,2,*], Florian Wimmers[1], Prabhu S Arunachalam[1], Wei Luo[3], Christopher B. Fox[4], Mark Tomai[5], Purvesh Khatri[1,2,#], Bali Pulendran[1,6,7,#]

[1]Institute for Immunity, Transplantation and Infection, Stanford University School of Medicine, Stanford University, Stanford, CA, USA.

[2]Center for Biomedical Informatics, Department of Medicine, Stanford University School of Medicine, Stanford, CA, USA.

[3]Department of Microbiology and Immunology, Indiana University School of Medicine, Indianapolis, IN, USA.

[4]Infectious Disease Research Institute, Seattle, WA, USA.

[5]3M Corporate Research and Materials Lab, St. Paul, MN, USA.

[6]Department of Pathology, Stanford University School of Medicine, Stanford University, Stanford, CA, USA.

[7]Department of Microbiology and Immunology, Stanford University School of Medicine, Stanford University, Stanford, CA, USA.

*These authors contributed equally.

# Correspondence to:
Bali Pulendran (bpulend@stanford.edu)
Purvesh Khatri (pkhatri@stanford.edu)

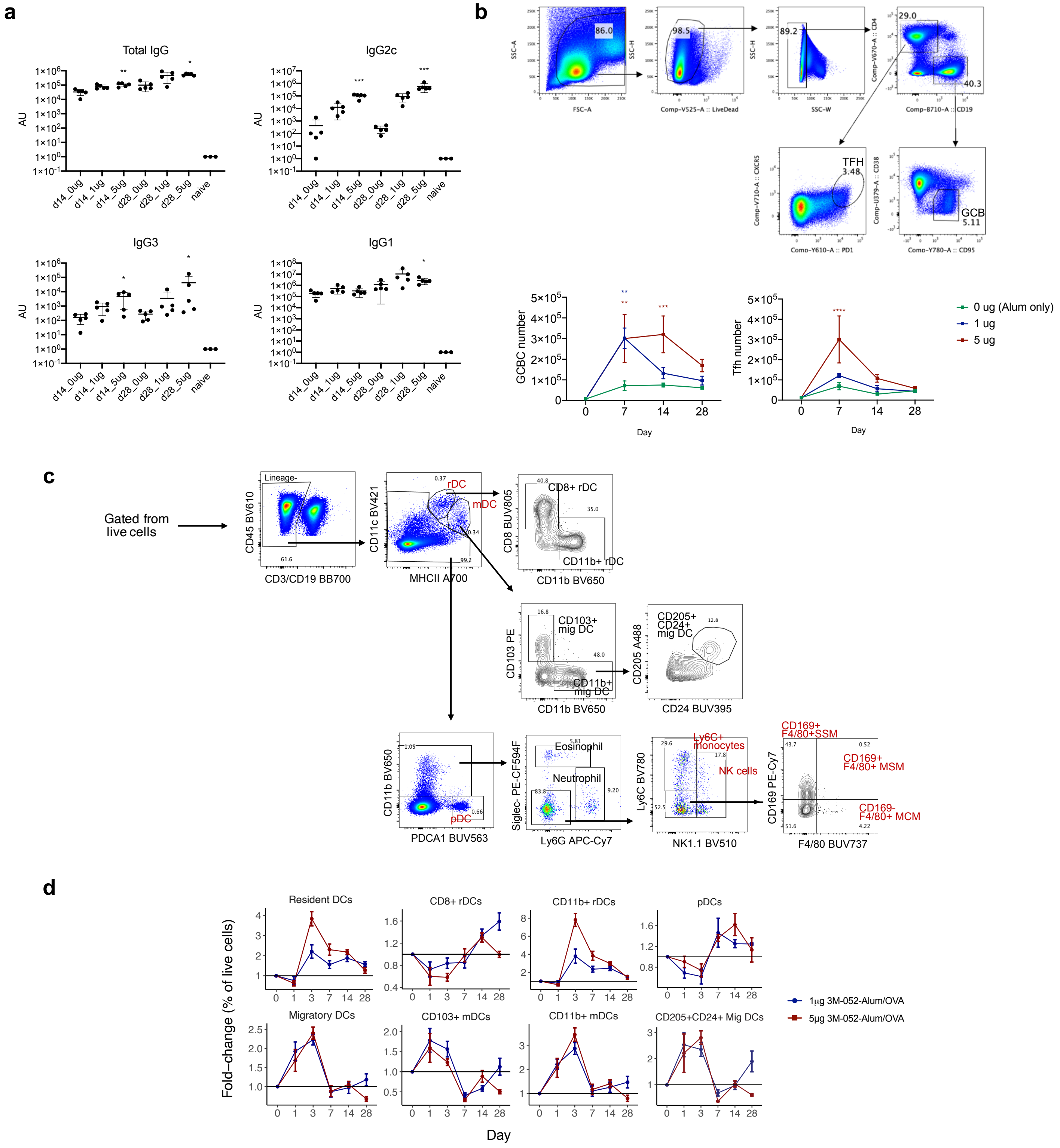

Supplementary Figure 1. **Humoral and innate responses in LNs following immunization. (a)** Antibody titers on day 14 and 28 post-immunization with indicated doses of 3M-052-Alum/OVA. One-way ANOVA with Dunn's multiple comparison test was performed against Alum only group at each timepoint (n=5 per group, n=3 in naïve group). **(a)** T follicular helper cell and germinal center B cell kinetics in draining lymph nodes over 28 days post-immunization with indicated doses of 3M-052-Alum/OVA. Two-way ANOVA with Tukey's multiple comparison test was performed (n=5 per group per timepoint; n=4 in 5ug group at day 7). **(c)** Gating strategy of innate cell populations. **(d)** Increase of DCs on day 3 is mainly constituted by CD11b+ rDCs and migratory DC subsets. Fold-change of mean % live cells ±SEM is represented (n=5 for each group; naïve group pooled from 3 independent experiments, n=15).

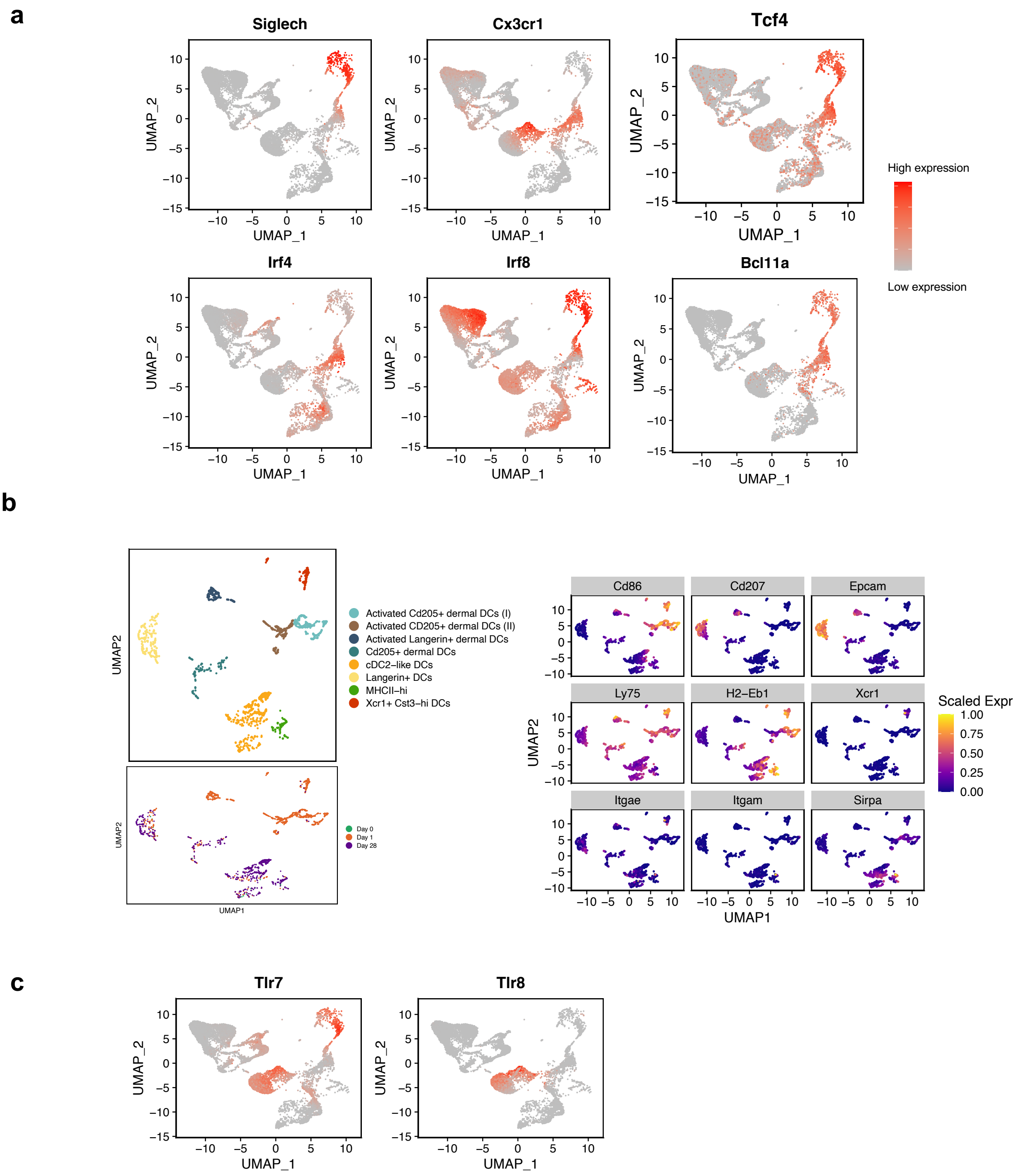

Supplementary Figure 2. Extended data on transcriptomics of 3M-052-Alum/OVA immunization. (a) Expression of genes previously found to represent tDC cluster shown on UMAP embedding of 3M-052-Alum/OVA scRNA-seq. **(b)** Migratory DC subclusters identify distinct migratory DC subsets in the dLN at all timepoints. **(c)** Gene expression of *Tlr7* and *Tlr8* in 3M-052-Alum/OVA scRNA-seq.

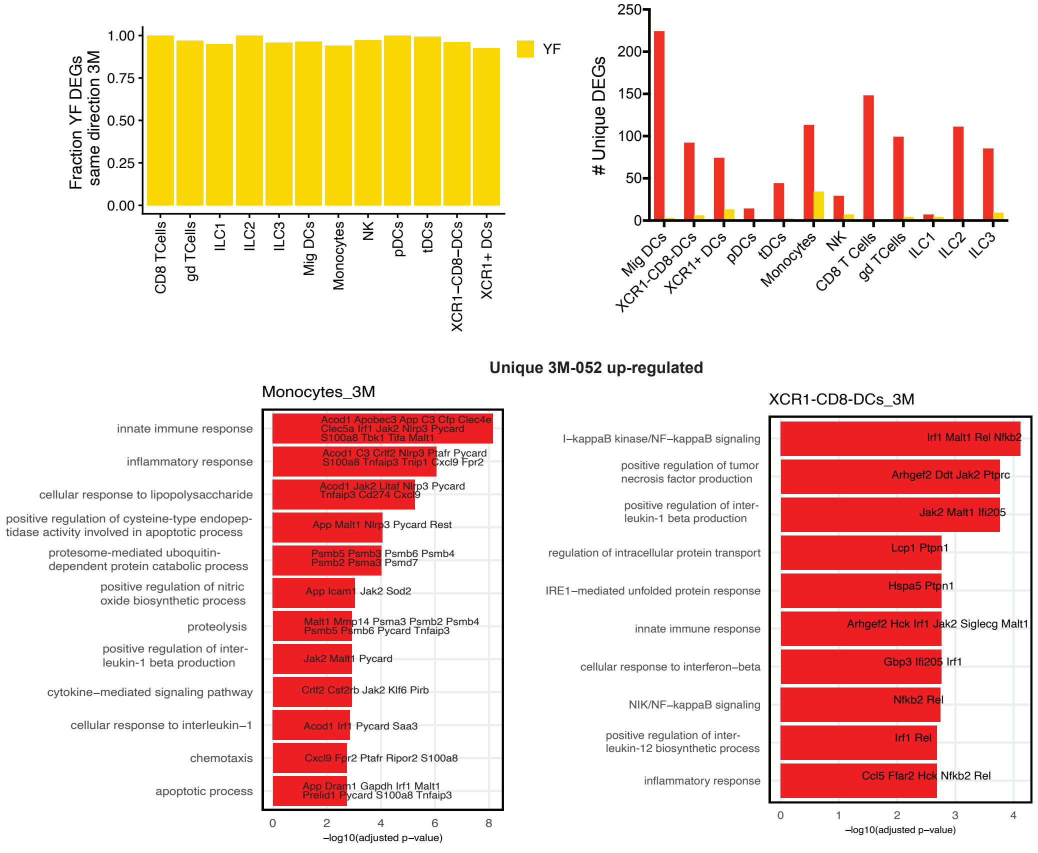

Supplementary Figure 3. Comparison of differential gene expression between 3M-052-Alum/OVA and YF-17D. Percentage of shared DEGs and number of unique DEGs (absolute logFC > 0.25, FDR < 0.05 and in opposite direction in the other group) across cell types in 3M-052 and YF-17D groups (top row). Enrichment of GO pathways of unique DEGs at day 1 post-immunization with 3M-052 (bottom row). Adjusted p-values are calculated using hypergeometric test with Benjamini-Hochberg correction.

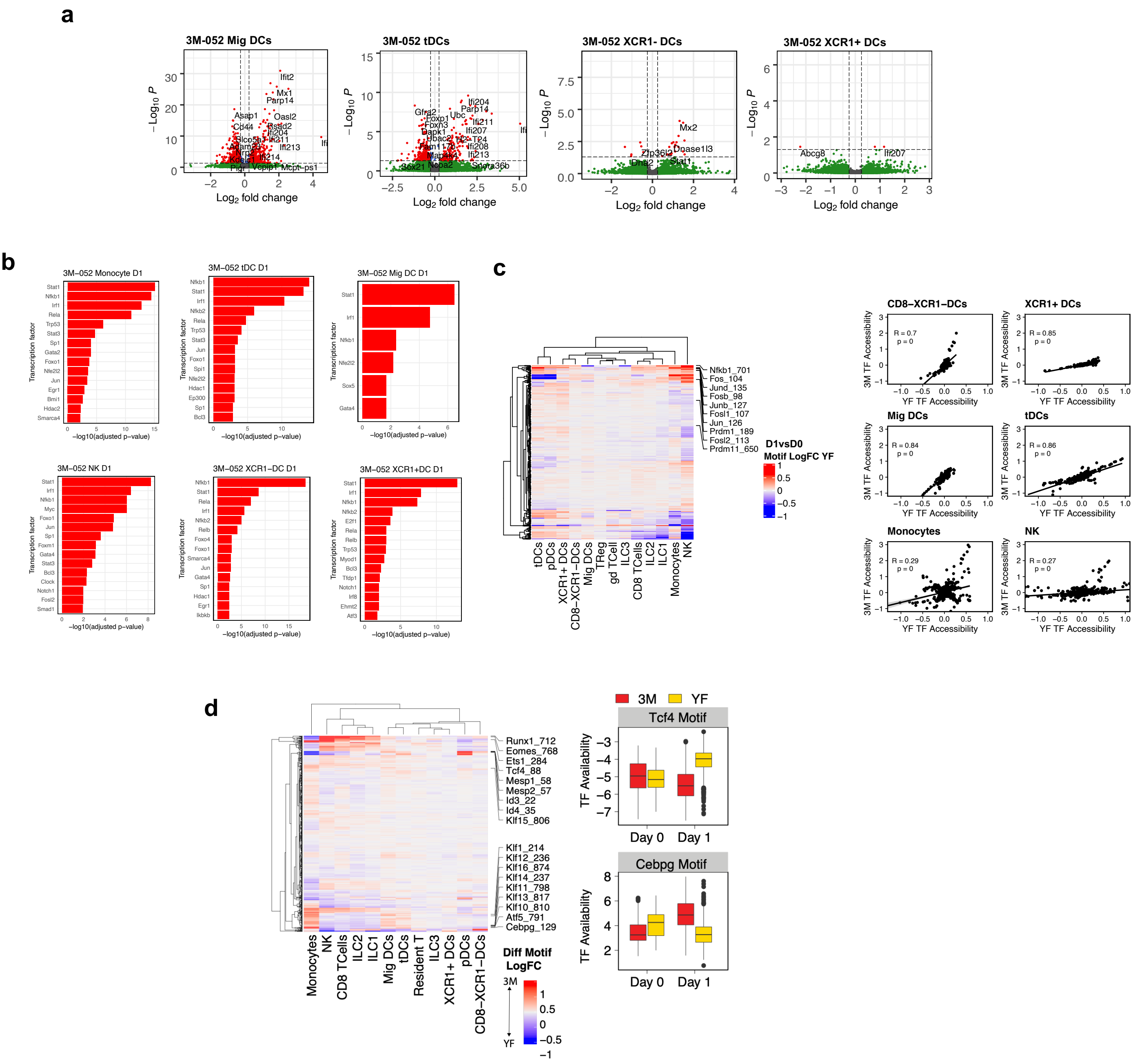

Supplementary Figure 4. Extended data on scATAC-seq following 3M-052-Alum/OVA immunization. (a) Volcano plots of day 1 differentially accessible genes after 3M-052-Alum/OVA immunization from scATAC-seq. Adjusted p-values are calculated using two-sided t-test with Benjamini-Hochberg correction. **(b)** Overrepresentation analysis of Day 1 DEGs using TRRUST TF-gene database. Adjusted p-values are calculated using hypergeometric test with Benjamini-Hochberg correction. **(c)** Day 1 logFC of TF motifs after YF-17D immunization from scATAC-seq. Correlation plots of TF motif accessibility across cell subsets between 3M-02-Alum/OVA and YF-17D groups. R represents Pearson's *r* and p represents two-tailed *P* values. **(d)** Heatmap of the difference of accessibility after 1 day in 3M-052-Alum/OVA compared to YF-17D. Each tile in the heatmap is calculated as 3M-052-Alum/OVA logFC – YF17D logFC for all TFs across each cell types (left). Boxplot with representative TF motifs that are differentially accessible only after YF-17D immunization (right) (top, Tcf4) or after 3M-052-Alum/OVA immunization (bottom, Cebpg) (3M052 monocytes, Day 0: n=419, Day 1: n=2380; YF-17D monocytes, n= Day 0: n=285, Day 1: n=3667). Center line of boxplot corresponds to the median, the bounds are the 75th and 25th percentiles (interquartile range; IQR), and the whiskers are the largest or smallest value no further than 1.5*IQR from the bounds.

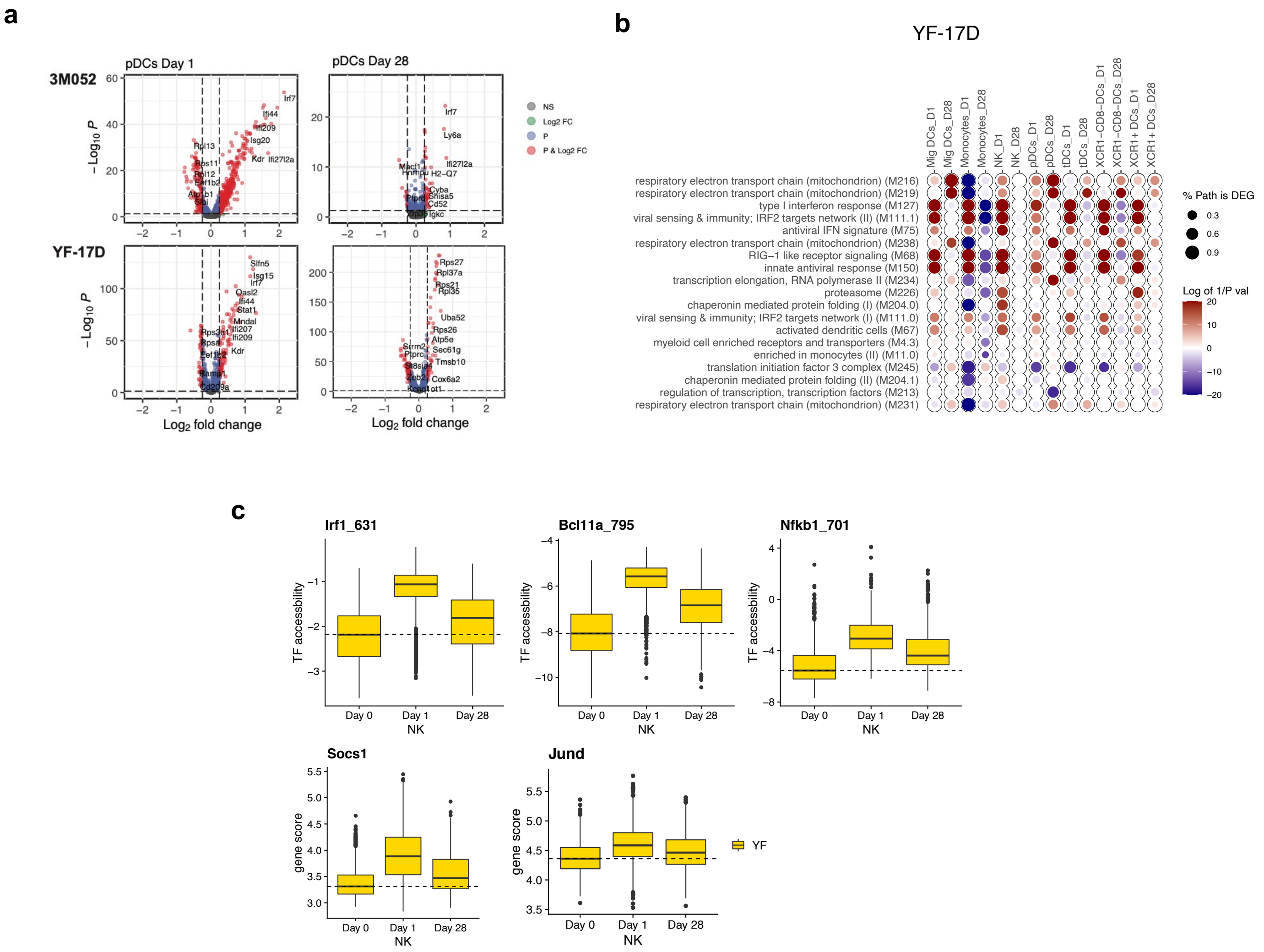

Supplementary Figure 5. Extended data on scRNA-seq and scATAC-seq following YF-17D immunization. (a) Differentially expressed genes in LN pDCs at day 1 and 28 post-immunization with 3M-052-Alum/OVA and YF-17D. Adjusted p-values are calculated using two-sided t-test with Benjamini-Hochberg correction. **(b)** Significantly enriched BTMs (FDR < 0.001) from overrepresentation of DEGs across innate cells on 1 and 28 days post-immunization with YF-17D. Adjusted p-values are calculated using hypergeometric test with Benjamini-Hochberg correction. **(c)** Boxplot of residual chromatin openness in Irf1, Nfkb, Bcl11a TF motif and *Jund* and *Socs1* genes in NK cells on day 0, 1, 28 after YF-17D immunization. (Day 0: n=1249, Day 1: n=6241, Day 28: n=1585). Center line of boxplot corresponds to the median, the bounds are the 75th and 25th percentiles (interquartile range; IQR), and the whiskers are the largest or smallest value no further than 1.5*IQR from the bounds.

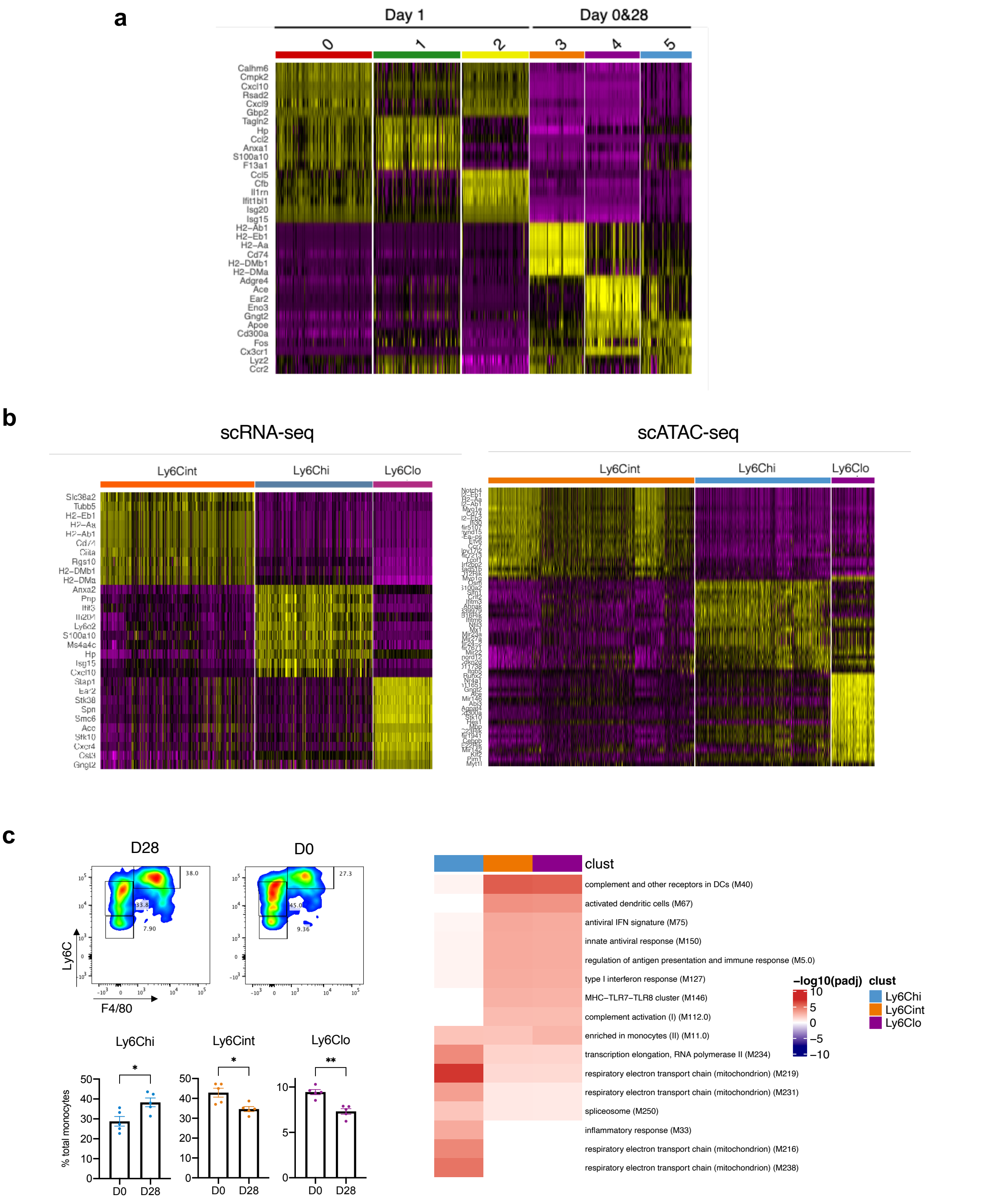

Supplementary Figure 6. Extended data on monocyte sub-clusters. (a) Gene signatures of day 1, 0 and 28 monocyte clusters in scRNA-seq.**(b)** Gene signatures of day 0 and 28 monocyte subclusters in scRNA-seq (left) and scATAC-seq (right). **(c)** Kinetics and dynamics of monocyte sub-clusters at day 28 and 0. % of Ly6C monocytes stained by flow cytometry at day 0 and 28 in LNs; two-tailed Mann-Whitney test (*, p-value=0.0317; **, p-value < 0.0079) (left). Overrepresentation analysis of differentially expressed genes (pval < 0.01, log2FC > 0.1) in monocyte subclusters (right). (n=5 per group). Mean ± SEM is represented.
