# Supplementary material for: A molecular atlas of innate immunity to adjuvanted and live attenuated vaccines, in mice
Source: Nat Commun. 2022 Jan 27;13:549. doi: 10.1038/s41467-022-28197-9 (PMC8795432; doi:10.1038/s41467-022-28197-9)
Supplement: Supplementary file 2 — Reporting summary [file 41467_2022_28197_MOESM2_ESM.pdf]

nature portfolio

Corresponding author(s): Bali Pulendran, Purvesh Khatri

Last updated by author(s): Dec 5, 2021

Reporting Summary

Nature Portfolio wishes to improve the reproducibility of the work that we publish. This form provides structure for consistency and transparency in reporting. For further information on Nature Portfolio policies, see our Editorial Policies and the Editorial Policy Checklist.

Statistics

For all statistical analyses, confirm that the following items are present in the figure legend, table legend, main text, or Methods section.

n/a	Confirmed	
☐	☒	The exact sample size (*n*) for each experimental group/condition, given as a discrete number and unit of measurement
☐	☒	A statement on whether measurements were taken from distinct samples or whether the same sample was measured repeatedly
☐	☒	The statistical test(s) used AND whether they are one- or two-sided<br>*Only common tests should be described solely by name; describe more complex techniques in the Methods section.*
☒	☐	A description of all covariates tested
☐	☒	A description of any assumptions or corrections, such as tests of normality and adjustment for multiple comparisons
☐	☒	A full description of the statistical parameters including central tendency (e.g. means) or other basic estimates (e.g. regression coefficient) AND variation (e.g. standard deviation) or associated estimates of uncertainty (e.g. confidence intervals)
☐	☒	For null hypothesis testing, the test statistic (e.g. *F*, *t*, *r*) with confidence intervals, effect sizes, degrees of freedom and *P* value noted<br>*Give P values as exact values whenever suitable.*
☒	☐	For Bayesian analysis, information on the choice of priors and Markov chain Monte Carlo settings
☒	☐	For hierarchical and complex designs, identification of the appropriate level for tests and full reporting of outcomes
☐	☒	Estimates of effect sizes (e.g. Cohen's *d*, Pearson's *r*), indicating how they were calculated

Our web collection on statistics for biologists contains articles on many of the points above.

Software and code

Policy information about availability of computer code

Data collection	Flow cytometry data were collected using BD FACS Diva v.8.01 software associated with BD FACS Symphony.
Data analysis	Flow cytometry data analyzed with FlowJo software v.10.7.1 (Treestar Inc) and GraphPad Prism version 9.0.0;<br>scRNA-seq data was analyzed in R version 4.0.2, using packages Seurat v 3.1.4, ComplexHeatmap v.2.1.0, Matrix v1.3, gridExtra 2.3, harmony 0.1.0, data.table 1.14.2, dplyr v1.0.7, cowplot v1.1.1, MetaIntegrator, igraph v1.2.6<br>scATAC-seq data was analyzed in R version 4.0.2, using packages Archr 1.0.1.<br>All statistical analysis are two-sided. All correlations are Pearson's R.

For manuscripts utilizing custom algorithms or software that are central to the research but not yet described in published literature, software must be made available to editors and reviewers. We strongly encourage code deposition in a community repository (e.g. GitHub). See the Nature Portfolio guidelines for submitting code & software for further information.

Data

Policy information about availability of data

All manuscripts must include a data availability statement. This statement should provide the following information, where applicable:

- Accession codes, unique identifiers, or web links for publicly available datasets
- A description of any restrictions on data availability
- For clinical datasets or third party data, please ensure that the statement adheres to our policy

scRNA-seq and scATAC-seq data are publicly accessible in the Gene Expression Omnibus under accession numbers GSE180384 and GSE180752, respectively. Codes are available at https://github.com/scottmk777/3M052Vaccine.

Field-specific reporting

Please select the one below that is the best fit for your research. If you are not sure, read the appropriate sections before making your selection.

- [x] Life sciences
- [ ] Behavioural & social sciences
- [ ] Ecological, evolutionary & environmental sciences

For a reference copy of the document with all sections, see nature.com/documents/nr-reporting-summary-flat.pdf

Life sciences study design

All studies must disclose on these points even when the disclosure is negative.

Sample size	No statistical test was used to predetermine the number of samples. Sample sizes were determined as appropriate for detecting significant vaccine effect in mouse studies performed by us and others and are commonly used sample sizes for similar experiments in the literature.
Data exclusions	No data were excluded from any of the analysis.
Replication	The assay was performed in one independent experiment with multiple biological replicates. Single cell experiments were performed in one independent experiment with biological replicates pooled together.
Randomization	All animals used in the study were age- and gender-matched.
Blinding	All the experiments were conducted in an unblinded way since the investigators were involved in overall conduct of the study. This is consistent with common practice in the field with studies of this nature.

Reporting for specific materials, systems and methods

We require information from authors about some types of materials, experimental systems and methods used in many studies. Here, indicate whether each material, system or method listed is relevant to your study. If you are not sure if a list item applies to your research, read the appropriate section before selecting a response.

Materials & experimental systems

n/a	Involved in the study	
[ ]	[x]	Antibodies
[x]	[ ]	Eukaryotic cell lines
[x]	[ ]	Palaeontology and archaeology
[ ]	[x]	Animals and other organisms
[x]	[ ]	Human research participants
[x]	[ ]	Clinical data
[x]	[ ]	Dual use research of concern

Methods

n/a	Involved in the study	
[x]	[ ]	ChIP-seq
[ ]	[x]	Flow cytometry
[x]	[ ]	MRI-based neuroimaging

Antibodies

Antibodies used

Antibody color dilution clone Cat. no.
CD205 FITC (A488) 1:100 205yekta eBioscience #53-2051-82
Ly6C BV780 1:500 HK1.4 Biolegend #128041
Ly6G APC-Cy7 1:400 1A8 Biolegend #127624
CD19 BB700 1:100 1D3 BD #566411
CD3 BB700 1:100 17A2 BD #742175
MHCII AF700 1:400 M5/114.15.2 eBioscience #56-5321-82
CD11b BV650 1:300 M1/70 Biolegend #101239
CD11c BV421 1:400 N418 Biolegend #117330
CD86 A647 1:300 GL-1 Biolegend #105020
Siglec-F PE-CF594 1:400 E50-2440 BD #562757
CD24/CD86 BUV395 1:200 M1/69 BD #744471
CD45 BV610 1:200 30-F11 Biolegend #103140
CD169 PE-Cy7 1:200 3D6.112 Biolegend #142412
PDCA-1 BUV563 1:200 927 BD #749275
CD8a BUV805 1:200 53-6.7 BD #612898
CD103 PE 1:100 2E7 eBioscience #12-1031-82
NK1.1/CX3CR1 BV510 1:200 PK136 Biolegend #108738
F4/80 BUV737 1:100 T45-2342 BD #749283
Zombie UV™ BUV496 1:250 Biolegend #423107

Ghost Dye™ Violet 510 BV510 1:400 Tonbo Bioscience #13-0870-T100
CD95 PE-Cy7 1:200 Jo2 BD #557653
CD19 PerCP-Cy5.5 1:200 1D3/CD19 Biolegend #152406
CD38 BUV395 1:200 90 Biolegend #102702
CD4 BV650 1:100 GK1.5 Biolegend #100469
CXCR5 BV711 1:50 L138D7 Biolegend #145529
PD1 PE-Dazzle594 1:200 29F.1A12 Biolegend #135228

Validation	All antibodies used were evaluated for the species (mouse) and application (flow cytometry) by the manufacturers as provided on their websites.

Animals and other organisms

Policy information about studies involving animals; ARRIVE guidelines recommended for reporting animal research

Laboratory animals	C57BL/6, 8-14 weeks old, female mice from Jackson Laboratory were used in this study. Mice were housed under specific-pathogen-free condition with standard 12:12 light:dark light cycles. Room temperature was maintained between 20 °C and 26 °C and humidity was maintained between 30 and 70%.
Wild animals	Study did not involve wild animals.
Field-collected samples	Study did not involve field-collected samples.
Ethics oversight	All animal experimentation and procedures were approved by Stanford University Institutional Animal Care and Use Committee.

Note that full information on the approval of the study protocol must also be provided in the manuscript.

Flow Cytometry

Plots

Confirm that:

- [x] The axis labels state the marker and fluorochrome used (e.g. CD4-FITC).
- [x] The axis scales are clearly visible. Include numbers along axes only for bottom left plot of group (a 'group' is an analysis of identical markers).
- [x] All plots are contour plots with outliers or pseudocolor plots.
- [x] A numerical value for number of cells or percentage (with statistics) is provided.

Methodology

Sample preparation

Lymph node processing and flow cytometry:
Inguinal lymph nodes were harvested from mice in PBS+2% FBS and treated with 5mg/ml collagenase type IV (Worthington) for 20 minutes at 37C, followed by smashing with a 100 micron strainer to obtain single cell suspension. Samples were then stained with flow cytometry antibodies for 20 minutes at 4C. Samples were washed twice with PBS + 2% FBS + 1mM EDTA and fixed with BD Cytofix (#554655).

Magnetic isolation and FACS sorting of LN cells:
Inguinal lymph nodes were harvested and processed to obtain single cell suspension. Total LN cells were then stained with anti-CD16/32 antibodies for blocking of Fc receptors, followed by staining with biotinylated anti-CD3 (Biolegend #100244) and anti-CD19 (eBioscience #13-0193-82) antibodies for 20 minutes at 4C. Next, samples were incubated with streptavidin conjugated magnetic beads (BD #557812) for 30 minutes at 4C. and passed through a magnet (STEMCELL technologies), according to manufacturer’s protocol. The negative fraction was transferred into a clean tube and stained for FACS sorting.

Instrument	BD FACS Symphony, FACSAria Fusion instrument
Software	Data were acquired using BD FACS Diva v.8.01 and the data were analyzed using FlowJo software v.10.7.1.

Cell population abundance

4 populations were sorted at each timepoint. The cell abundance was determined by cell number sorted on FACSAria Fusion instrument and are as follows:
For scATAC-seq:
cell_type timepoint 3M052_count YF_count
cd11b+pdca1+ naïve 20600 53200
cd11b+pdca1+ d01 95400 238000
cd11b+pdca1+ d28 19500 65000
pdcs (pdca1+) naïve 1300 16200
pdcs d01 35600 86600
pdcs d28 14000 27100

dcs (CD11c+ MHCII+) naïve 23800 -
dcs d01 59600 211000
dcs d28 35800 97500
ly6c+ naïve 22800 5420
ly6c+ d01 92000 363000
ly6c+ d28 17320 16200

For scRNA-seq:
cell_type timepoint 3M052_count YF_count
cd11b+pdca1+ naïve 9600 20300
cd11b+pdca1+ d01 91000 98800
cd11b+pdca1+ d28 16200 29800
pdcs (pdca1+) naïve 46600 133000
pdcs d01 44400 58300
pdcs d28 35800 92000
dcs (CD11c+ MHCII+) naïve 25000 265000
dcs d01 30400 214000
dcs d28 41200 211000
ly6c+ naïve 41000 36500
ly6c+ d01 34600 47500
ly6c+ d28 25000 25800

4-way purity was used for sorting and all sorted samples have a purity of > 80% as determined by the efficiency recorded on the sorter.

Gating strategy

Cells were selected based on FSC-A vs SSC-A, singlets were selected using FSC-A vs FSC-H. Live CD3/CD19- cells were used for gating of innate immune cells. CD11chi MHC-II int cells were selected as rDCs and CD11cint MHCII hi cells were selected as mDCs. CD11c- cells were separated into CD11b- PDCA1+ pDCs and CD11b+ cells.

☒ Tick this box to confirm that a figure exemplifying the gating strategy is provided in the Supplementary Information.
